# Intestinal Nematode Infection Affects Metastasis of EL4 Lymphoma Cells

**DOI:** 10.1007/s00005-020-00594-2

**Published:** 2020-09-22

**Authors:** Katarzyna Donskow-Łysoniewska, Katarzyna Krawczak, Maja Machcińska, Klaudia Brodaczewska

**Affiliations:** 1 Laboratory of Parasitology, General Karol Kaczkowski Military Institute of Hygiene and Epidemiology, Warsaw, Poland; 2 Laboratory of Molecular Oncology and Innovative Therapies, Military Institute of Medicine, Warsaw, Poland

**Keywords:** Immunomodulation, Intestinal nematodes, Tumour, Metastasis

## Abstract

An effective host immune system prevents the growth of most cancer cells. However, as intestinal nematodes are able to induce both immunotolerance and immunosuppression in the host, it is possible that their presence could allow co-occurring cancer cells to proliferate and metastasize. Our findings indicate that previous, subsequent or concurrent intestinal nematode infection affects the formation of lung metastatic nodules in mice experimentally infected with *Heligmosomoides polygyrus*. In addition, pre-infection with nematodes renders mice resistant to metastasis development in lungs, with the inoculated EL4 cancer cells being located mainly in mesenteric lymph nodes. The present paper discusses the nematode-induced mechanisms which may influence the metastatic process.

## Introduction

Parasite infections induce a wide range of adaptive changes in the host, ranging from changes in the immune system, through physiological changes, to behavioural changes (Wraith 2017). In addition, factors produced and released by the parasite are known to induce immunoregulatory mechanisms that can inhibit the inflammatory reaction (Maizels et al. 2004). Such immunosuppression and immunotolerance probably arose during the course of host-parasite coevolution with the aim of protecting both the host and the parasite against the effects of excessive inflammatory reactions; during this time, the immunogenicity of many parasitic antigens has tended to decrease, while the parasitic mechanisms regulating the host immune response have evolved and become stronger (Garside et al. 2000). Consequently, many studies in recent years have proposed the use of helminth therapy and helminth-derived product therapy based on helminths such as *Trichuris trichiura* and *Necator americanus* as potential treatment for autoimmune diseases. However, nematode colonization can be potentially life threatening in more susceptible patients, such as those with compromised or suppressed immune systems (Correale 2014).

To survive for a long time in an adverse and aggressive environment, invading nematodes secrete a range of soluble factors that may modify host-cell homeostasis by interacting with host cells (Donskow et al. 2011; Donskow-Łysoniewska et al. 2013; Packham and Stevenson 2004). In addition, both parasites and tumours provoke an immune response and promote persistent growth in surrounding and peripheral tissue, and both have developed numerous methods to avoid detection and eradication by the immune system. One such strategy involves the recruitment of infiltrating leukocytes, such as immunoregulatory myeloid cells, regulatory T (Treg) cells CD8^+^ and CD4^+^, T helper 17 cells and regulatory B cells, and modulating their expansion and function. These cells have been found to protect parasite and cancer cells from the immune response and relieve the stresses associated with the hostile environment in the host (Dunn et al. 2004; Maizels et al. 2004).

One prominent factor that is often found to be overproduced in animal models of cancer, cancer patients and in cases of parasite infection is transforming growth factor (TGF)-β. TGF-β is produced by *inter alia* T cells, and has wide-ranging effects on tumour or parasite development: it promotes primary tumour growth and tumour metastasis by suppressing anti-tumour T cell function, suppresses the immune response to maintain worm burden through pathways involving TGF-β production, and maintains worm fecundity by influencing TGF-β activity (Doligalska et al. 2006; Li and Flavell 2008). Furthermore, in the absence of the host source of TGF-β in physiological settings, nematodes can act on mammalian receptors and activate the TGF-β pathway by proteins that functionally mimic the immunosuppressive TGF-β (Johnston et al. 2017).

In addition, TGF-β plays a crucial role in maintaining the tolerance of peripheral T cells to self-antigens.

Accordingly, infection may change or even improve the tumour microenvironment, and influence the immune responses of the host and immunomodulatory functions of the tumour. The question of whether intestinal nematode infection can contribute to tumour development and metastasis remains an interesting yet unexplored avenue of research. Understanding the precise effects of nematode infection on tumours is of fundamental interest in developing safe, targeted helminth-derived therapies. To address this question, the present study examines the effect of intestinal nematode infection on the development of malignancy under fully-controlled experimental conditions. The intestinal nematode chosen for the experiment was *Heligmosomoides polygyrus*: a member of the Trichostrongylidae family which takes part in natural, asymptomatic, chronic invasions in mice. During infection, *H. polygyrus* inhibits inflammatory reactions by similar mechanisms to those employed by a fellow strongylid, the hookworm *Necator americanus*, in humans; this similarity is not surprising considering the high proteomic and genomic homology between the two (Moreno et al. 2011). To address the current lack of information in the literature regarding this aspect of nematode-host relations, the present paper examines the effect of *H. polygyrus* nematode infection on EL4, a lymphoblast cell line that secretes TGF-β.

## Materials and Methods

The study group was formed from male C57BL/6 mice, aged between 8 and 10 weeks (weight 22–27 g). All mice were allowed to adjust to laboratory conditions for seven days at the animal-house facilities before the study. They were housed in cages in groups of six, at a temperature of 24–25 °C, 50% humidity and a 12-h/12-h lighting regimen. Food and water were provided ad libitum. All mice were maintained under specific pathogen-free conditions, and animal experimentation was conducted according to the rules of the Local Ethics Committee.

The EL4 asyngenic murine lymphoma cells were cultured in vitro in RPMI-1640 media supplemented with 10% foetal calf serum, 0.1 mM glutamine and 10 U/ml penicillin. The cells were then washed two times in endotoxin-free PBS pH 7,2 and labelled with carboxyfluorescein succinimidyl ester (CFSE, eBioscience, San Diego, CA, USA). Following this, 1 × 10^6^ cells were injected intravenously to the mice through the tail vein in a volume of 0.1 mL PBS pH 7.2. Cell viability was estimated to be at least 95% according to trypan blue exclusion.

*H. polygyrus* infective larvae (L3) were prepared in drinking water. Briefly, the mice were infected per os with 300 L3 using a gavage tube. Following the experimental protocol, the mice were terminally anesthetized and sampled individually. Adult nematode numbers were estimated using the Baermann technique.

The EL4 T-lymphoma-bearing C57BL/6 mice were divided into groups of six. Each group followed one of three protocols: (a) the mice were infected with *H. polygyrus* in drinking water 15 days after EL4 cell inoculation (EL4-Hpoly); a set of controls were also formed that received drinking water without nematodes (EL4). (b) The mice were infected with *H. polygyrus* in drinking water, 15 days before inoculation with EL4 cells (Hpoly-EL4); a group of controls was also set up that only received drinking water (EL4″). (c) The mice were infected with *H. polygyrus* and inoculated with EL4 cells at the same time (EL4&Hpoly).

All animals were weighed and observed daily. Fifteen (EL4&Hpoly and Hpoly-EL4) or 30 days (EL4-Hpoly) from the start of experiment, the mice were terminally anesthetized with O_2_/CO_2_. The isolated lungs were fixed in Bouin’s fixative and the number of metastases counted and measured with a dissecting microscope.

Blood samples (700–800 μL) were taken from all mice by intracardiac puncture. The samples were treated with heparin, and the resulting serum was isolated and stored at − 80 °C. The mesenteric lymph nodes (MLN) and spleen were isolated aseptically from the mice and pressed through a nylon cell strainer (BD, Falcon) to produce a single**-**cell suspension. The blood was depleted of erythrocytes by hypotonic lysis and washed in PBS pH 7.2.

Lung tissue samples were prepared by mechanical disruption followed by 1-h treatment with 0.5 mg/mL collagenase Type D (Sigma-Aldrich, St. Louis, MO, USA) at 37 °C. Digested tissue was pressed through a cell strainer (0.2 μm) (BD, Falcon) to produce a single**-**cell suspension. The cells were washed and re-suspended in PBS pH 7.2. The viability of cells were ascertained using Muse Count and Viability Assay kits (Merck Millipore, USA) using a Muse Cell Analyzer (Merck Millipore, USA) in accordance with the manufacturer’s instructions.

The MLN, spleen and lungs were examined for EL4 CFSE-positive cells, and the CFSE dilution was determined by FACSCalibur (BD Biosciences, San Jose, CA) and analysed by FACS Diva software Version 6.1.2. Live lymphocytes were manually gated on the basis of forward scatter and side scatter. Immunofluorescence reactivity was determined by flow cytometry set to analyse 500,000 cells in the sample gate.

Serum TGFβ-1 concentration was measured in serum and in EL4 cell culture after 24 h by ELISA (e-Biosciences, San Diego, USA) according to the manufacturer’s recommendations. The plates were read at 490 nm. The readings were taken in triplicate, and the mean optical densities (OD) were compared with the standard curves prepared using recombinant proteins.

Results were analysed with a one-way analysis of variance (ANOVA) or Student’s *t* test with Statistica software. Differences between groups were considered significant when the *p* < 0.05. Significant relationships are denoted with an asterisk on graphs. All experimental groups comprised six mice kept in one cage, and all experiments were performed in triplicate, yielding similar results.

## Results

**Fig. 1:**
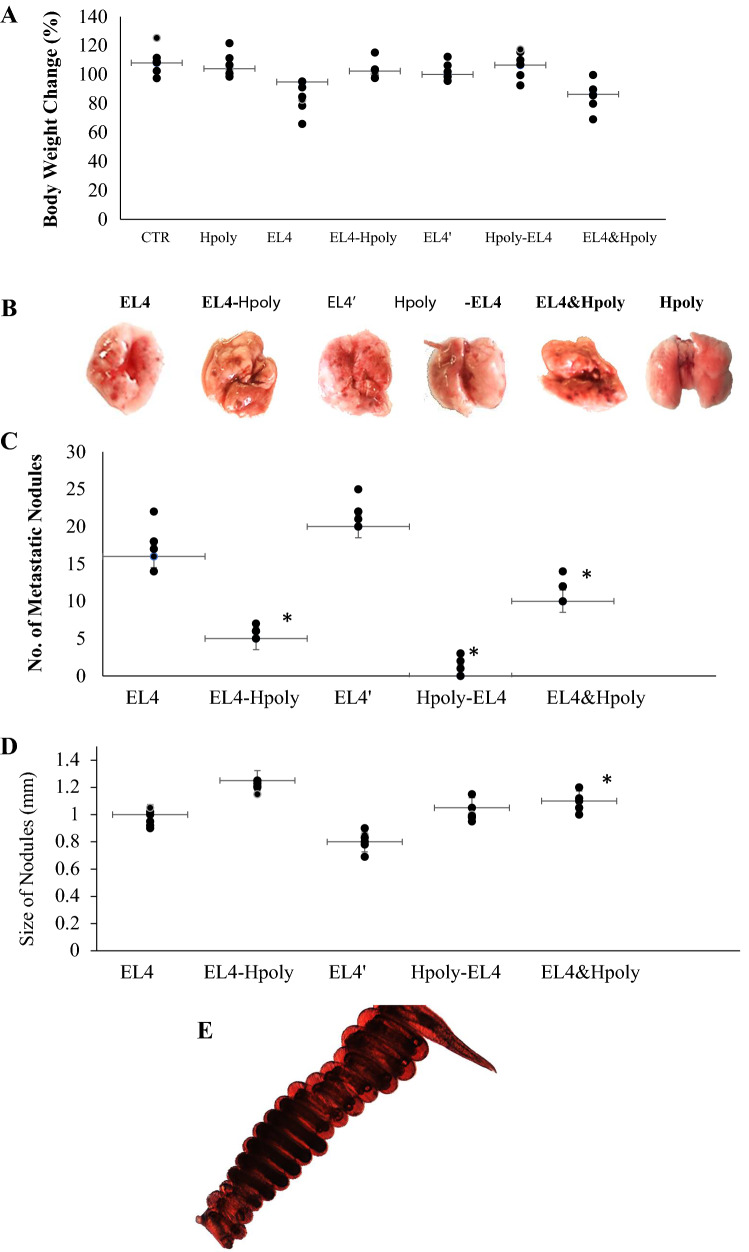
Experimental metastasis studies using EL4 cell line in C57BL6/J mice infected with the intestinal nematode *H. polygyrus*. Mice were injected with either 1 × 10^6^ EL4 cells or PBS pH 7.2 (vehicle control) and infected with nematodes, as described in “Materials and Methods”. The mice were observed daily for changes in body weight. Final changes in body weight are presented as (**a**). After 15–30 days, the mice were sacrificed and the lungs excised and photographed (**b**). Pulmonary metastatic nodules were assessed according to number and size (**c**, **d**). *H. polygyrus* female morphology (**e**). Data are representative of three independent experiments. Six animals were used in each experimental group. Error bars (horizontal/vertical) indicate standard errors (SE), and an asterisk indicates a significant difference (*p* < 0.05) to analogous control mice injected with EL4 cells

The effect of nematode infection on EL4 metastasis was examined in three experimental models. All mice were inoculated with EL4; however, one set of mice was post-treated with the nematode, one was pre-treated with the nematode and another was co-treated with the nematode. Although no significant differences in the body weight were observed between the uninfected controls and those that were pre- or post-infected with the nematode, a significant increase was observed in the co-infected mice (EL4&Hpoly group, Fig. 1a). No significant difference was observed between the parasite burdens of analysed groups during infection.

In the control mice, the inoculated EL4 cells grew progressively, leading to detectable tumours in lungs (EL4) at 15 days post inoculation and massive contiguous lung metastases at 30 days post inoculation (EL4′). Nematode infection had significant effects on tumour metastasis: significantly fewer lung EL4 tumours were observed in mice infected with nematodes. In mice pre-infected with *H. polygyrus,* lung metastases were almost completely rejected, while two of the six showed no signs of tumour growth in the lungs: it would appear that pre-infection appears to induce resistance to tumour development in lungs; however, tumour nodules were observed in the MLN (data not shown).

**Fig. 2:**
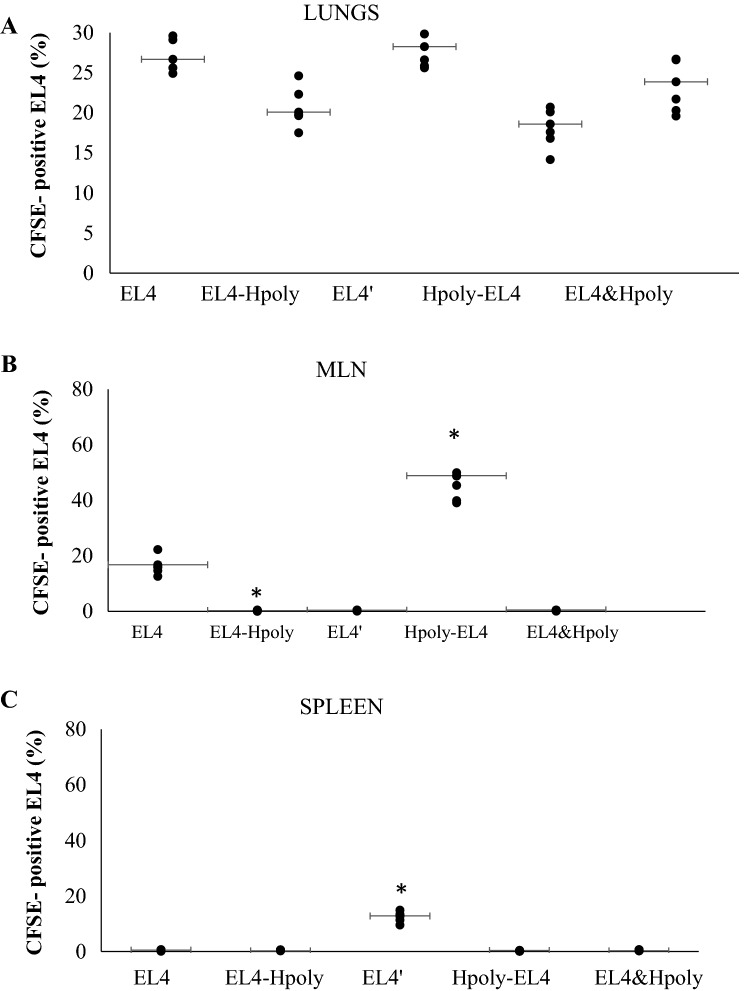
EL4 cell colonization in C57BL6/J mice infected with *H. polygyrus* intestinal nematodes. Flow cytometric analysis examining the percentage of EL4 carboxyfluorescein succinimidyl ester (CFSE)-labelled cells in the lungs (**a**), MLN (**b**) and spleen (**c**) of mice. For each group, the analysis was performed on individual cells isolated from individual mice, error bars indicate standard errors (SE), and an asterisk indicates a significant difference (*p* < 0.05) to analogous control mice injected with EL4 cells

In the early and later stages following EL4 inoculation (15–30 days), a large number of small-sized EL4 nodules were observed in controls (0.8 mm). Those observed in all groups of mice infected with nematodes were significantly larger (1.3 mm; Fig. 2b–d). The pre-infected mice (Hpoly-EL4) presented fewer nodules in the lungs; however, their presence was accompanied by visible nodules in the MLN (data not shown).

The locations of the transferred CFSE-stained EL4 cells were determined based on the presence of various malignant nodules in mice with nematode infections*.* The presence of CFSE-positive trafficked lymphocytes in the single-cell suspensions from the lungs, spleen and MLN were evaluated by flow cytometry.

Significantly greater numbers of EL4 cells were detected in the lungs of control mice (EL4 and EL4′) than in those of the mice infected with nematodes (Fig. 2).

Similar percentages of CFSE-positive cells were observed in pre-infected mice, co-infected mice and controls; however, the pre-infected mice demonstrated lower numbers of CFSE-labelled cells in the lungs, and these cells were trafficked to the MLN, where nodules were observed (Fig. 2): almost 45% of the MLN cells of the pre-infected mice were CFSE-positive (Fig. 2b). No changes in CFSE-positive cells were observed in the spleen for all mice infected with nematodes (Fig. 2c). No nodules were found in any other organs or in the peritoneal cavity.

**Fig. 3:**
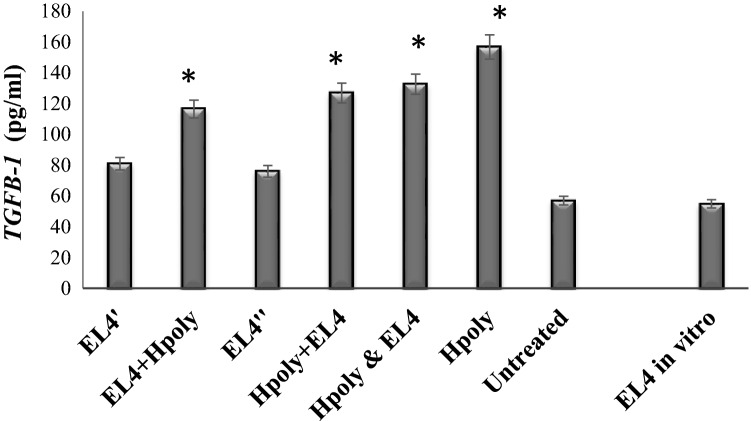
Effect of nematode infection on serum *TGFβ-1* concentration in mice transferred with lymphoma EL4 cells*.* Data are representative of three independent experiments. Six animals were used in each experimental group. Error bars indicate standard errors (SE), and an asterisk indicates a significant difference (*p* < 0.05) to analogous control mice injected with EL4 cells

The EL4 cells produced TGFβ-1 in vitro, and inoculation with EL4 led to elevated TGFβ-1 levels in serum. *H. polygyrus* infection intensified such elevation in all tested groups of mice (Fig. 3).

## Discussion

The immunomodulatory status associated with nematode infection influences both dividing and non-dividing cells, and it is possible that the factors secreted by helminths could play a role in the promotion and progression of neoplasms. *Schistosoma haematobium, Taenia taeniaformis, Spiromera mansonoides* and *T. solium* possess significant tumour-promoting activity (Herrera and Ostrosky-Wegman 2001). In addition, excretory-secretory products from the small intestine nematodes *Trichostrongylus vitrinus, Trichostrongylus colubriformis, Cooperia curticei* and *Nematodirus battus*, and the abomasal nematode *Teladorsagia circumcincta*, have all been shown to induce over-proliferation in normal intestinal epithelial cells and cell lines (Huby et al. 1995), and exposure to Anisakis larvae has recently been reported as a potential risk factor for gastric and colon adenocarcinoma (Garcia-Perez et al. 2015). However, the precise nature of the influence exerted by nematodes on the immune response to tumours remains unknown. In the present study, our aim was to identify how intestinal nematode infection influences the infiltration of EL4 cancer cells and the subsequent formation of metastatic foci in lungs in vivo.

EL4 cell lines with inherent immunogenicity and the capacity to form metastatic tumours following injection were established. The established EL4 aggressive tumour lines are syngeneic to C57BL6 mice.

To determine the influence of *H. polygyrus* infection on the frequency of metastasis of lung carcinomas from bone preferential sites, mice inoculated with EL4 cells were divided into three groups: one was pre-infected with *H. polygyrus*, another was co-infected at the same time as inoculation, and the third was post-infected. The EL4 cells formed intensive contiguous lung metastases by 15–30 days post inoculation. No decrease in body weight was found to be associated with EL4 lymphoma induction, indicating that EL4 nodule loading does not induce obvious signs of disease, expressed as significant changes in body weight, and that intestinal nematode burden also does not affect body weight.

In addition, *H. polygyrus* infection did not appear to exacerbate the condition, indicating that it is does not play an essential role in the induction of host tolerance to EL4 metastasis. Although lower numbers of lung nodules were detected in the pre-infected and co-infected mice, these were of larger size than those of the EL4 control mice. Comparable percentages of trafficked EL4 cells were observed in the lungs of groups of infected mice, suggesting that subsequent and concurrent infection is important for nodule formation.

The differences in nodule size observed between infected mice and controls suggest that the nodules may be formed by different pathways, either by the division of a single EL4 cell, or from a number of different cells. Although nematode infection significantly reduced the numbers of cancer cell colonies, the presence of large nodules might reflect the proliferative abilities and aggressive potential of cancer cells.

Interestingly, a lower frequency of lung metastases was observed in mice previously infected with *H. polygyrus*, suggesting that pre-infection of mice with nematode renders them resistant to tumour development in lungs. After injection of EL4 cells, the cancer cells were transported to specific organs via the blood, and tumour nodules were found growing in the MLN. Therefore, in this group of mice, lung colonization of EL4 would not appear to be a definitive measure of host protective immunity.

The inhibition of host immune responses might be realised by multiple mechanisms, with Treg lymphocytes and myeloid-derived suppressor cells being the potential target of nematodes. Treg cells produce various regulatory cytokines, such as interleukin 10 and TGF-β, and directly influence other leukocytes. Treg cells were shown to block the activation of dendritic cells by influencing the expression of co-stimulatory molecules, thus hampering their maturation. Myeloid-derived suppressor cells directly and indirectly suppress antigen-specific and non-specific activation of CD4^+^ and CD8^+^ lymphocytes (Maizels et al. 2004; Valanparambil et al. 2017).

In vivo, EL4 tumour line growth is associated with TGF-β production in both the tumour and host cells (Gorelik and Flavell 2001). Our results confirm that EL4 cells produce TGFβ-1 in vitro, that nematode infection enhanced the concentration of TGF-β in mouse serum in vivo, and that this elevation was associated with different forms of metastatic foci in the lungs. TGFβ-1 is enhanced during *H. polygyrus* infection (Doligalska et al. 2006). *H. polygyrus* secretes a ligand which activates the TGF-β pathway, providing an example of structural mimicry which replicates the functional activity of this cytokine (Johnston et al. 2017; Smyth et al. 2018). However, it was postulated that the suppressive effect of Treg cells on EL4 cells in vitro works in a dose-dependent manner through cell–cell contact, but not in a cytokine-dependent manner (Ying et al. 2009). Further studies are necessary to explore the possibility that TGF-β mimics a protein on EL4 cells. TGF-β plays a dual role in tumour development: both promoting cancer progression and suppressing the development of tumours in spontaneous cancer (Pu et al. 2009).

To conclude, our findings indicate that intestinal nematode infection was associated with elevated TGFβ-1 levels and resulted in fewer, but larger, tumour nodules in the lungs. The molecular background of this phenomenon is not yet known, but it is evident that the changes in the metastatic microenvironment induced by nematode infection affects the immunogenic or immunomodulatory abilities of the host. The functional outcome of T cell responses to tumours is governed by a balance between anti-tumour responses and inhibitory signals. It is possible that changes caused by the nematode infection are directed at tumour cells and could indirectly regulate Treg cell activity or angiogenesis.

The present study has certain limitations. Firstly, the immune responses to transplanted tumours could be fundamentally different from those associated with spontaneous tumours (Willimsky and Blankenstein 2007), and further experimental studies are needed to confirm this. In addition, our system did not reflect the reality of the first stage of cancer metastasis in terms of migration of cells to a primary site for infiltration and subsequent growth based on their escape and mobility; however, our findings nevertheless provide an indication of the changes provoked by nematode infection. Finally, as both nematodes and EL4 secrete TGF-β, it remains unclear which source has the greatest influence on metastasis formation, or how it may be affected by increased numbers of transferred EL4 cells. The area of research needs further study: the development of safe and trustworthy helminth therapy for inflammatory disorders and cancer immunotherapy in humans remains one of the most important and scientifically interesting challenges faced by modern medicine.
